# Prévalence des hépatites virales B et C chez les hommes ayant des rapports sexuels avec d’autres hommes mobilisés pour une étude de démonstration de la prophylaxie préexposition au VIH à Cotonou, au Bénin

**DOI:** 10.11604/pamj.2023.46.79.41013

**Published:** 2023-11-09

**Authors:** Luc Béhanzin, Souleymane Diabaté, Fernand Aimé Guédou, Ella Goma Matsétsé, Marius Olodo, Alban Dossouvo, Marlène Aza-Gnandji, Axel Akpaka, Elyote Chagas, Flore Gangbo, Djimon Marcel Zannou, Michel Alary

**Affiliations:** 1 École Nationale de Formation des Techniciens Supérieurs en Santé Publique et en Surveillance Epidémiologique, Université de Parakou, Parakou, Bénin,; 2 Organisation pour la Promotion de la Santé et le Développement Communautaire, Cotonou, Bénin,; 3 Dispensaire IST, Centre de Santé Communal de Cotonou 1, Cotonou, Bénin,; 4 Centre de Recherche du CHU de Québec-Université Laval, Québec, Canada,; 5 Département de Médecine Sociale et Préventive, Université Laval, Québec, Canada,; 6 UFR Sciences Médicales, Université Alassane Ouattara, Bouaké, Côte d´Ivoire,; 7 Bénin Synergie Plus, Cotonou, Bénin,; 8 Réseau Sida Bénin, Cotonou, Bénin,; 9 Programme Santé de Lutte contre le Sida, Cotonou, Bénin,; 10 Faculté des Sciences de la Santé, Université d´Abomey-Calavi, Cotonou, Bénin,; 11 Centre National Hospitalier Universitaire HMK, Cotonou, Bénin,; 12 Institut National de Santé Publique, Québec, Canada

**Keywords:** Hommes ayant des rapports sexuels avec d´autres hommes, Infections sexuellement transmissibles, hépatite B, hépatite C, hépatite B actuelle, hépatite B à vie, facteurs associés, prévalence, Men who have sex with men, sexually transmitted infections, hepatitis B, hepatitis C, associated factors, prevalence, Cotonou, Benin

## Abstract

**Introduction:**

les hommes ayant des rapports sexuels avec d´autres hommes (HSH) sont touchés de façon disproportionnée par le virus de l´hépatite B (VHB) et le virus de l´hépatite C (VHC) dans le monde. Au Bénin où il n´y a pas de données sur les HSH, cette étude visait à estimer les prévalences du VHB et du VHC et les facteurs associés au VHB chez des HSH séronégatifs au virus de l´immunodéficience humaine (VIH).

**Méthodes:**

dans cette étude transversale à visée analytique, un échantillonnage aléatoire à deux degrés a permis de recruter 204 HSH. Un test immunochromatographique rapide (One Step Multi-infectious Disease Test) et des tests immuno-enzymatiques (Monolisa) ont été utilisés pour détecter antigènes/anticorps du VHB et du VHC. La régression log-binomiale a été utilisée pour l´identification des facteurs associés au VHB.

**Résultats:**

les prévalences de l´AgHBs, d´antécédent d´hépatite B et de l´hépatite C étaient, respectivement, de 8,8%, 37,7% et 0,9%. L´AgHBs et l´antécédent d´hépatite B étaient plus fréquents chez les HSH de 30 ans et plus comparativement aux plus jeunes: 16,7% contre 6,4% (p<0,0001) et 66,7% contre 28,8% (p<0,0001), respectivement. Les rapports sexuels sous l´effet de la drogue ou de l´alcool et le fait de vivre en couple étaient aussi associés à l´hépatite B.

**Conclusion:**

la prévalence de l´hépatite C était faible, mais l´hépatite B était fréquente, surtout chez les HSH plus âgés. Le dépistage et la vaccination contre l´hépatite B devraient être renforcés au sein de cette population.

## Abstract (en)

**Introduction:**

men who have sex with men (MSM) are disproportionately affected by hepatitis B virus (HBV) and hepatitis C virus (HCV) worldwide. In Benin, there are no data on MSM. The purpose of this study was to estimate HBV and HCV prevalence and HBV-associated factors in MSM who were HIV negative.

**Methods:**

we conducted an analytical cross-sectional study. A two-degree random sampling was used to recruit 204 MSM. An immunochromatographic rapid test and enzyme immunoassays were used to detect HBV and HCV antigens/antibodies. Log-binomial regression was used to identify factors associated with HBV.

**Results:**

HbsAg positivity, history of hepatitis B infection and hepatitis C prevalences were 37.7%, 8.8 %, and 0.9 %, respectively. HBsAg positivity and history of hepatitis B were more prevalent in MSM aged ≥30 years compared to younger subjects: 16.7% versus 6.4% (p<0.0001) and 66.7% versus 28.8% (p<0.0001), respectively. Sexual intercourse under the effect of drug or alcohol and living in couple were also associated with HBV.

**conclusion:**

the prevalence of hepatitis C was low, but hepatitis B was common, especially among older MSM. Screening and vaccination against hepatitis B should be strengthened in this population.

## Introduction

Selon l´Organisation Mondiale de la Santé (OMS), l´hépatite B et C causeraient, respectivement, 1,5 (Intervalle de confiance à 95%, IC95%: [1,1-2,6]) et 1,5 (IC95%: [1,3-1,8]) million de nouvelles infections chaque année dans le monde [1]. La forme chronique de ces infections est l´une des principales causes de la cirrhose et du cancer primitif du foie qui sont responsables des décès [1-3]. Depuis 2017, les hépatites virales notamment celles B et C sont considérées par l´OMS comme la quatrième priorité de santé publique à l´échelle mondiale et plus particulièrement dans la région africaine, après l´infection due au virus de l´immunodéficience humaine (VIH), le paludisme et la tuberculose [4]. Plus de 70% des hépatites estimées à travers le monde surviennent en Afrique [5]. Au Bénin, en 2013, chez les nouveaux donneurs de sang, la prévalence de l´hépatite B était de 9,9% et celle de l´hépatite C de 4,1 [6]. En 2014, le nombre global d´infections et de décès liés aux hépatites B et C au Bénin était respectivement estimé à 1,3 million et 340 000 [7]. En 2016, dans la population générale, la prévalence de l´hépatite B était de 6,7% et celle de l´hépatite C de 2,5%, ce qui faisait du Bénin une zone à endémicité élevée pour ces deux infections [6]. Le virus de l´hépatite B (VHB) et le virus de l´hépatite C (VHC) sont essentiellement transmis par voie parentérale. La contamination se fait par voie sexuelle (le virus de l'hépatite B est de 50 à 100 fois plus contaminant que le VIH), à travers l´usage de drogues (aiguilles ou seringues contaminées), la transfusion de produits sanguins contaminés, les tatouages et piercings sans précautions d´asepsie [8,9]. Il existe un vaccin contre l´hépatite B avec une efficacité vaccinale après trois doses de 100% chez le nourrisson et de 95% chez le jeune adulte [8]. Le vaccin Pentavalent contenant l´antigène vaccinal contre l´hépatite B a été intégré au calendrier vaccinal du programme élargi de vaccination au Bénin en 2005 et la première dose est actuellement administrée dans les 24 premières heures de la naissance [10]. L´hépatite C n´a pas encore de vaccin homologué; sa prévention repose essentiellement sur le dépistage systématique des anticorps anti-VHC lors des dons du sang, et l'utilisation de matériel à usage unique [8]. Les hommes ayant des rapports sexuels avec d´autres hommes (HSH) sont affectés de manière disproportionnée par les hépatites B et C à cause de certains facteurs comme le multi-partenariat sexuel et l'usage des drogues injectables [8,11,12]. A ce jour, au Bénin, aucune étude n'a été faite sur la prévalence et les facteurs associés à l´hépatite B et C chez les HSH. La présente étude, qui vise à combler ce déficit de connaissances, a pour objectif d´estimer la prévalence des hépatites B et C et d´identifier les facteurs associés à l´hépatite B au sein des HSH séronégatifs au virus de l´immunodéficience humaine (VIH) à Cotonou au Bénin.

## Méthodes

**Cadre de l´étude**: l'étude a été conduite à Cotonou, la capitale économique de la république du Bénin, un pays d'Afrique de l'Ouest. Sa population était estimée à 679 012 habitants selon le recensement général de la population de 2013 avec projection à 2 401 067 d'habitants en 2017 [13]. À Cotonou, les HSH étaient organisés en deux grands réseaux, Réseau Bénin Synergie Plus (BeSyP) et Réseau Sida Bénin (RSB). Le dispensaire IST (DIST) de Cotonou, la plus grande clinique de dispensation des services adaptés aux populations clés du VIH dont les HSH, a servi de cadre de recrutement des participants à l´étude.

**Type d´étude**: il s'est agi d'une étude transversale à visée analytique ayant utilisé les données de la phase de recrutement des HSH dans l´étude de démonstration à base communautaire de la prophylaxie préexposition (PrEP) à l'infection au VIH, une étude de cohorte prospective, 2020-2021 (PrEP-HSH) [14].

**Population d´étude**: l'étude a ciblé Les HSH de la ville de Cotonou en 2020. La population source était constituée des HSH appartenant à l´un des réseaux des HSH à Cotonou. Une étude de cartographie des HSH faite par BeSyP en 2017 a rapporté une taille de 3391 HSH à Cotonou [15].

**Critères d´inclusion**: pour participer à l'étude, il faut être: a) un HSH né de sexe masculin; b) être âgé d'au moins de 18 ans; c) être membre d´un des réseaux des HSH du Bénin (BeSyP et RSB); d) avoir donné son consentement écrit libre et éclairé de participer à l´étude; e)être confirmé séronégatif pour le VIH au Dispensaire IST de Cotonou, Bénin.

**Critères d´exclusion**: a) Était exclu de l'étude: b) tout HSH qui pour une raison ou une autre ne peut donner son consentement écrit vraiment libre et éclairé (maladie invalidante notamment mentale ou pour toute autre cause); c) tout participant à l´étude qui, pour une raison quelconque, a refusé de compléter le circuit de recrutement dans l´étude PrEP-HSH.

**Échantillonnage et taille minimale de l´échantillon**: la conception de la présente recherche était intégrée au protocole de l'étude PrEP-HSH. Notre étude transversale a utilisé les données de la phase de recrutement dans ladite étude [14]. Les participants potentiels ont été sélectionnés suivant un sondage aléatoire à deux degrés. Au premier degré, sur les 13 arrondissements que comptait la commune de Cotonou, sept ont été sélectionnés selon une probabilité proportionnelle à la taille et suivant la technique des totaux cumulés. Au second degré, la méthode du parcours aléatoire a été utilisée dans chaque arrondissement sélectionné [16]. Ce parcours aléatoire a été réalisé par 10 HSH animateurs formés dans le cadre de l´étude PrEP-HSH. Nous avons utilisé la formule de Schwartz pour estimer la taille minimale de l'échantillon. Selon Adeyemi *et al*., de façon générale, il y a une très grande variabilité de la prévalence de l´hépatite B au sein des HSH [17]. L´étude de Adeyemi au Nigeria a rapporté une prévalence de 10% [6,50%-13,53%] au sein des HSH à sérologie négative au VIH [18]. En supposant la prévalence populationnelle de 13,53% de cette étude, avec une précision de 5%, nous avons estimé à 180 la taille minimale de l'échantillon. L'étude ayant utilisé 204 HSH pour la prévalence de l´hépatite B et les facteurs qui y étaient associés, elle garantissait alors une puissance suffisante pour la détection des différences statistiques significatives existantes.

**Figure 1:**
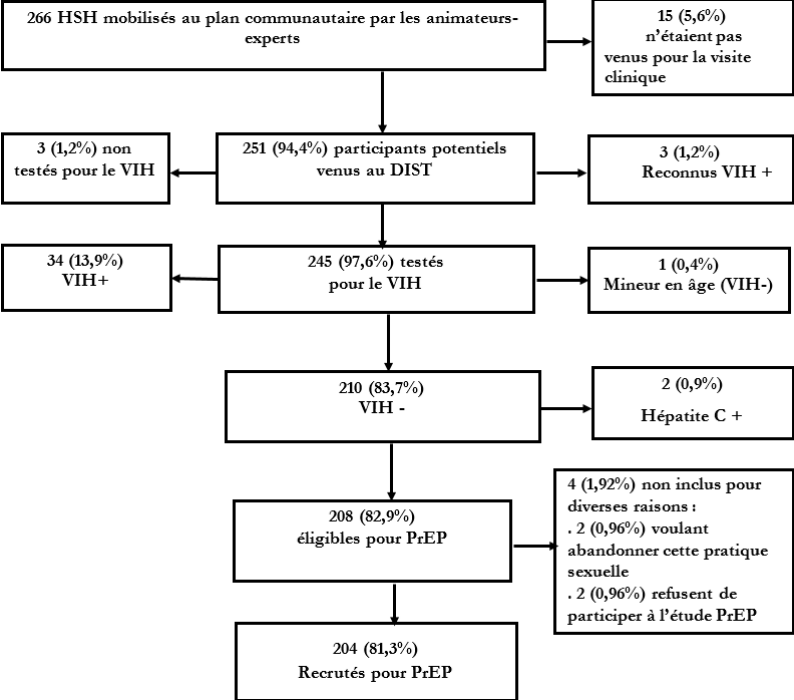
diagramme de flux de recrutement des participants dans l’étude de démonstration PrEP au VIH chez les HSH à Cotonou, Bénin, 2020-2021

**Recrutement des participants et collecte des données**: le processus du parcours aléatoire de l´échantillonnage a été réalisé par 10 pairs-animateurs HSH dits animateurs experts, car spécifiquement formés pour l´étude PrEP-HSH. Ces animateurs orientaient les participants potentiels au DIST, la clinique de recrutement et du suivi dans l´étude PrEP. Les HSH s´identifiant comme séronégatifs mobilisés par les animateurs-experts, munis de coupon de mobilisation communautaire de la cible, étaient reçus au DIST selon leur ordre d´arrivée. Sur la période de recrutement dans l´étude, les 10 animateurs-experts ont mobilisé et orienté au DIST, selon le plan d´échantillonnage, 266 HSH qui s´identifiaient comme séronégatifs au VIH. Parmi eux, 15 ne se sont pas rendus au DIST. Sur les 245 examinés pour l´éligibilité au DIST, 210 étaient séronégatifs au VIH. De ce nombre, six ont été exclus pour diverses raisons dont deux à cause de la présence de l'anticorps anti-VHC (l´hépatite C étant une contre-indication pour la PrEP orale utilisant la combinaison générique d´emtricitabine et de fumarate de ténofovir disoproxil, Figure 1). Ainsi, la collecte des données pour l´hépatite B et les facteurs associés a été réalisée chez 204 participants. Au DIST, la première étape était la validation de leur séronégativité au VIH par le: *« One Step Multi-infectious Disease Test »*. Un résultat non réactif à ce test signifiait l´absence du VIH tandis qu´un résultat réactif exigeait la réalisation d´un deuxième test de confirmation: « SD Bioline HIV-1/2 3.0 de Standard Diagnostics ». Lorsque les deux tests étaient réactifs, le HSH était déclaré porteur du VIH. Les échantillons de sang des HSH avec des résultats discordants (c'est à dire premier test réactif, deuxième test non réactif) étaient acheminés au laboratoire de référence du VIH pour la confirmation ou non de la présence du VIH par un test ELISA et/ou la recherche des acides ribonucléique/désoxyribonucléique du virus. Seuls les HSH séronégatifs pour le VIH étaient intégrés dans le circuit de recrutement pour l´étude PrEP-HSH. La collecte des données pour l´hépatite B et facteurs associés a été alors réalisée chez les 204 recrutés dans l'étude PrEP-HSH. Le recrutement s´est déroulé du 24 août au 24 novembre 2020. Une fois recrutés dans l´étude, les participants ont eu à répondre à un questionnaire face-à-face portant sur les caractéristiques sociodémographiques, leur état général de santé, leurs comportements sexuels, l´évaluation des risques d´acquisition et de transmission des IST et les symptômes d´infections sexuellement transmissibles. Le questionnaire a été digitalisé pour une collecte numérique grâce à l´application KoboCollect® qui est gratuite.

**Echantillons biologiques et tests de détection des marqueurs de l´hépatite B et des anticorps anti VHC**: la détection de l´Antigène de surface du VHB (AgHBs) et des anticorps anti-VHC a été réalisée à partir du sang capillaire (piqure au bout du doigt) au moyen d´un test immunochromatographique rapide « One Step Multi-infectious Disease Test » de la marque INTEC qui est une cassette de dépistage multiple (HBsAg/HCV/HIV/TP; InTec Products, Inc., Xinyang, China). Les anticorps dirigés contre le core (anti-HBc) et les protéines de surface du VHB (anti-HBs) étaient recherchés par des méthodes qualitatives quand l´AgHBs était absent. Cette recherche des anticorps anti-HBc et anti-HBs était réalisée, à partir d´un échantillon de plasma sanguin, au moyen de tests immono-enzymatiques Elisa (enzyme-linked immunosorbent assay): MonolisaTM Anti-HBs PLUS (#72566) et MonolisaTM Anti-HBcPLUS (#72315) de Bio-Rad Laboratories.

**Traitement et analyse des données**: les données collectées étaient transférées et stockées au fur et à mesure sur un serveur gratuit et sécurisé de l´UNOCHA (Bureau des Nations Unies pour la coordination de l´assistance humanitaire). Ensuite, elles ont été extraites sous format EXCEL et les analyses statistiques ont été réalisées par le logiciel STATA 17 (sataCorp LLC 4950 Lakeway Drive College Station, TX 77845, USA). Les caractéristiques des participants ont été décrites à l´aide de moyennes (écart-type) ou de médiane (intervalle interquartile, IIQ) pour les variables continues et de proportions assorties de leur intervalle de confiance à 95% pour les variables qualitatives. La comparaison des proportions a été faite avec le test Khi-deux de Pearson ou le test exact de Fisher, selon le cas. Le portage d´AgHBs a été défini par la détection de l´AgHBs au test rapide « One Step Multi-infectious Disease Test ». La proportion des participants ayant un antécédent d´hépatite B a été estimée par le total des participants chez qui on a détecté l´AgHBs ou l´anti-HBc (isolé ou associé à anti-HBs), c´est-à-dire ceux ayant fait une hépatite B ne serait-ce qu´une fois au cours de la vie. Le seuil de positivité du test Monolisa TM Anti-HBs PLUS utilisé pour la détection des anticorps anti-HBs était de 10 mUI/ml [19]. Le seuil de positivité du test Monolisa TM Anti-HBc PLUS utilisé pour la détection des anti-HBc était déterminé par calcul d´observance [20]. La régression log-binomiale a été utilisée pour l´identification des facteurs associés à l´hépatite B. Pour tous les tests statistiques, le seuil de signification statistique de 5% a été choisi.

**Considérations éthiques**: le Comité national d´éthique pour la recherche en santé du Bénin (No 014/MS/DC/SGM/DRFMT/CNERS/SA du 15 février 2020) et le comité d´éthique du CHU de Québec-Université Laval à Québec, Canada (Projet 2020-5106/Approbation finale du 20 avril 2020) ont approuvé le protocole d´étude. Tout s´est déroulé conformément aux autorisations reçues dans ces avis éthiques. La participation à l´étude était soumise à un consentement écrit libre éclairé. Les participants ont été informés du caractère confidentiel des données recueillies et de l'anonymat dans le traitement et les analyses de ces données. Les participants non vaccinés et sans hépatite B (AgHBs négatif) ont reçu une vaccination anti-hépatite B gratuite.

## Résultats

**Caractéristiques de la population d´étude**: l´âge moyen des 204 hommes était de 26,2 ±4,9 ans. La majorité (76,5%) parmi les HSH étaient âgés de moins de 30 ans. Près de la moitié (48%) d´entre eux avaient un niveau universitaire et étaient presque tous Béninois (95,6%). Le rôle sexuel préféré (63,1%) était insertif (Tableau 1).

**Tableau 1:** caractéristiques sociodémographiques et comportements sexuels des HSH au recrutement dans une étude de démonstration de la PrEP pour la prévention du VIH les ciblant, Cotonou, Bénin, 2020-2021

Caractéristiques sociodémographiques	n	%	IC 95%*
Catégories d’âges (année) (N=204)			
<20	7	3,4	1,4 - 6,9
20-24	77	37,7	31,1 - 44, 8
25-29	72	35,3	28,8 - 42,3
30-34	36	17,6	12, 7 - 23,6
≥35	12	6,0	3,1 - 10,1
Moyenne âge ± ET**	26,2 ±4,9		
Médiane âge (IIQ***)	25,0 (22,0 - 29.0)		
Pays d’origine (N=204)			
Bénin	195	95,6	91,8 – 98,0
Autres****	9	4,4	2,0 - 8,2
Religion (N=204)			
Traditionnel	11	5,4	2,7 - 9,4
Christianisme	167	81,9	75,9 - 86,9
Islam	26	12,7	8,5 - 18,1
Niveau le plus élevé d’éducation atteint (N=204)			
Aucun	1	0,5	0,0 - 2,7
Primaire	14	6,9	3,8 - 11,3
Secondaire (niveau 1)	34	16,7	11,8 - 22,5
Secondaire (niveau 2)	57	27,9	21,9 - 34,6
Supérieur	98	48,0	41,0 - 55,1
Situation matrimoniale (N=204)			
Marié	19	9,3	5,7 - 14,2
Célibataire	165	80,9	74,8 - 86,0
Divorcé	1	0,5	0,0 - 2,7
Separé	3	1,5	0,0 - 4,2
veuf	1	0,5	0,0 - 2,7
Vivant avec un partenaire	15	7,3	4,2 - 11,8
Rôle sexuel (N=187)			
Actif or insertif	118	63,1	55,8 - 70,0
Passif ou réceptif	39	20,9	15,3 - 27,4
Alternativement	30	16,0	11,1 - 22,1

* Intervalle de confiance à 95%; **Écart-type; ***Intervalle interquartile; ****Togo, Nigeria, Ghana, Cameroun

**Prévalence des hépatites**: la prévalence du portage de l´AgHBs et celle d´antécédent d´hépatite B étaient respectivement de 8,8% et 37,7% (Tableau 2). Aussi bien le portage d´AgHBs (16,7% contre 6,4%, p < 0,0001) que l´antécédent d´hépatite B (66,7% contre 28,8%, p < 0,0001) étaient plus prévalents chez les HSH âgés de 30 ans et plus comparativement aux plus jeunes (Tableau 2). La prévalence de l'hépatite C sur la base du multi-test était de 0,9% (2/210) (Figure 1). Les tests immuno-enzymatiques ont montré que 40 des HSH (19,6%) avaient une immunité résultant d´une hépatite B antérieure (anticorps Anti-HBc et Anti-HBs tous positifs, les seuils de positivité et d´immunité sont de 10 mUI/ml pour les Anti-HBs) [19], 19 (9,3%) étaient non immunisés contre l'hépatite B (anticorps Anti-HBc seul positif) et pouvaient avoir une infection en cours, être des personnes à risque, ou des personnes ayant guéri d'une infection,31 participants (15,2%) étaient déjà vaccinés contre l´hépatite B (présence isolée d´anticorps anti-HBs) et 96 (47,1%) n´étaient jamais entrés en contact avec le virus de l´hépatite B (Tableau 3). L´âge, les rapports sexuels sous l´effet de la drogue ou de l´alcool et le fait de vivre en couple étaient statistiquement et significativement associés au portage de l´AgHBs et à l´antécédent de l´hépatite B au seuil de signification statistique de 5% (Tableau 4).

**Tableau 2:** répartition du portage d’AgHBs et d’antécédent de l’hépatite B selon certaines caractéristiques des HSH au recrutement dans une étude de démonstration de la PrEP pour la prévention du VIH les ciblant, Cotonou, Bénin, 2020-2021

	Portage de l’AgHBS (Ag HBs +) N (%)				Antécédent d’hépatite B* N (%)			
	Oui	Non	Total	Valeur-p	Oui	Non	Total	Valeur-p
**Age**								
< 30	10 (6,4)	146 (93,6)	156 (100,0)	0,040***	45 (28,9)	111 (71,1)	156 (100,0)	< 0,0001**
≥ 30	8 (16,7)	40 (83,3)	48 (100,0)		32 (66,7)	16 (33,3)	48 (100,0)	
Total	18 (8,8)	186 (91,2)	204 (100,0)		77 (37,7)	127 (62,3)	204 (100,0)	
**Participant vivant avec un partenaire**								
Oui	9 (14,1)	55 (85,9)	64 (100,0)	0,074**	32 (50,0)	32 (50,0)	64 (100,0)	0,015**
Non	9 (6,4)	131 (93,6)	140 (100,0)		45 (32,1)	95 (67,9)	140 (100,0)	
Total	18 (8,8)	186 (91,2)	204 (100,0)		77 (37,7)	127 (62,3)	204 (100,0)	
**Sexe anal sous l’effet de drogue ou d’alcool**								
Oui	4 (40,0)	6 (60,0)	10 (100,0)	0,006***	7 (70,0)	3 (30,0)	10 (100,0)	0,044***
Non	14 (7,2)	180 (92,8)	194 (100,0)		70 (36,1)	124 (63,9)	194 (100,0)	
Total	18 (8,8)	186 (91,2)	204 (100,0)		77 (37,7)	127 (62,3)	204 (100,0)	

* AgHBs+ ou anti-HBc+ associé ou non à anti-HBs+

**Khi-deux de Person

*** Khi-deux exact de Fisher

**Tableau 3:** résultats des tests de sérologie d´immunisation contre l´hépatite B chez les HSH non porteurs d´AgHBs au recrutement dans une étude de démonstration de la PrEP pour la prévention du VIH les ciblant, Cotonou, Bénin, 2020-2021

Variables	Anti HBs- (N=204)	Anti HBs+ (N=204)	Total
Anti HBc-	96 (47,06%)	31 (15,20%)	127 (62,25%)
Anti HBc+	19 (9,31%)	40 (19,61%)	59 (28,92%)
**Total**	115(56,37%)	71(34,80%)	186(91,18%)

**Tableau 4:** facteurs associés au portage d’AgHBs et l’antécédent d’hépatite B chez les HSH au recrutement dans une étude de démonstration de la PrEP pour la prévention du VIH les ciblant, Cotonou, Bénin, 2020-2021

	Portage d’AgHBs (Ag HBs +) (N=204)			Antécédent d’hépatite B* (N=204)		
	RPA**	IC 95%***	Valeur-p	RPA	IC 95%	Valeur-p
**Age**						
< 30	1			1		
≥ 30	2,25	1,16 - 4,38	0,017	1,98	1,45 - 2,69	< 0,001
**Participant vivant avec un partenaire**						
Oui	2,5	1,2 - 5,3	0,018	1,5	1,1 - 2,0	0,004
Non	1			1		1
**Sexe anal sous l’effet de drogue ou d’alcool**						
Oui	6,4	3,4 - 12,1	< 0,001	1,8	1,4 - 2,2	< 0,001
Non	1			1		

* AgHBs+ ou anti-HBc+ associé ou non à anti-HBs+; **Rapport de prévalences ajusté, ***Intervalle de confiance à 95%

## Discussion

La prévalence du portage de l´AgHBs et celle de l´antécédent de l´hépatite B étaient respectivement de 8,8% et 37,7%, mais spécifiquement ces prévalences étaient significativement plus élevées chez les sujets âgés de 30 ans et plus comparativement aux plus jeunes. La prévalence de l´hépatite C était de 0,9%. Les HSH dans notre étude étaient majoritairement jeunes avec un âge moyen à 26,2 ±4,9 ans, et plus des trois quarts étaient âgés de moins de 30 ans. Ils étaient tous lettrés, sauf un seul participant qui n'avait aucun niveau d´éducation. La majorité (92%) des participants scolarisés avaient au moins un niveau d´éducation secondaire et 48% d´entre eux avaient le niveau universitaire. La structuration fortement juvénile observée dans la population des HSH a été retrouvée aussi dans l´étude de Loukabou *et al*. en 2019 auprès des HSH de Brazzaville et de Pointe-Noire en république du Congo (âge moyen à 28,8 ± 5,4 ans) [21]. La même observation a été faite dans l'étude de Rhodes *et al*. en 2021 à Abuja et Lagos au Nigeria où l'âge médian était de 25 ans (IIQ: 22 - 27) [18]. Dans l´enquête de surveillance de deuxième génération auprès des HSH au Bénin en 2022, 45,3% [41,3-49,2] des HSH étaient également âgés de 15 à 24 ans [22].

La prévalence du portage d´AgHBs de 8,8% [4,93% - 12,71%] observée dans notre étude n´est pas significativement différente de celle de 6,7% [6,5%-6,9%] retrouvée dans la population générale au Bénin en 2016 [6]. Cette homogénéité de risque de l'hépatite B chez les HSH au sein de la grande population pourrait s´expliquer par le fait que les HSH dans notre étude étaient tous de statut séronégatif au VIH et n´avaient pas de pratiques à risque de transmission parentérale de l´hépatite. En effet, aucun des HSH n´a rapporté consommer de drogue injectable. En général, pour la consommation de la drogue, sur les 204 HSH, 19 ont rapporté être fumeurs de marijuana. Dans l´étude en République du Congo à Brazzaville et à Pointe Noire, sur les 161 HSH interrogés, seulement deux ont déclaré avoir eu la pratique de consommation de drogue par voie injectable [21]. L'étude de Adeyemi a rapporté une prévalence du portage de l´AgHBs de 10% [6,650%-13,53%] au sein des HSH à sérologie négative au VIH, laquelle prévalence était semblable à celle observée dans notre présente étude [18]. La prévalence plus élevée chez les plus âgés pourrait s´expliquer par le fait que la vaccination contre l´hépatite B en combinaison dans le vaccin Pentavalent n´était intégrée au programme élargi de vaccination (PEV) au Bénin qu'en 2005 (06 semaines, 10 semaines et 14 semaines de la naissance) sans rattrapage [10], donc aucun de nos participants les plus âgés n´a bénéficié de cette vaccination contre l'hépatite intégrée au PEV. De plus, il y a eu l´effet de cumul dans le temps pour l´âge, des déterminants comportementaux d´acquisition de l´infection au virus de l'hépatite B. On pourrait penser aussi au démarrage tardif des interventions ciblant cette population des HSH au Bénin, par le Plan International- Bénin en 2008, après que les plus âgés aient commencé leurs pratiques de trans-orientation sexuelle. À Abuja et Lagos au Nigeria, la prévalence du portage de l´AgHBs qui était plus élevée chez les plus jeunes de 16 à 20 ans pouvait s´expliquer par le fait que l'échantillon était constitué de 61% de HSH à sérologie positive pour le VIH [18]. En effet, la prévalence de l´hépatite B est plus élevée chez les personnes vivant avec le VIH, les deux infections partageant le même mode de transmission [23,24]

Dans notre étude, plus de 48% des HSH ont un niveau d´instruction universitaire. Ce qui est similaire à la proportion retrouvée dans l´étude de l’INSAE où 34% des HSH recrutés en 2015 avaient un niveau universitaire en considérant l'effet de tendance croissante des niveaux d´étude dans le temps comme ce fut le cas de l´enquête démographique et de Santé au Bénin en 2017-2018 comparativement à celle de 2012 [25]. Même en considération de cet effet de tendance croissante dans les niveaux d'instruction, la proportion des HSH en 2020 ayant un niveau universitaire était plus élevée que celle des hommes de la population générale au Bénin et qui était de 7% en 2018 [25]. Dans notre étude, 96 participants (47,06%) n´étaient jamais entrés en contact avec le virus de l´hépatite B et leur vaccination était nécessaire. Nous n´avons pas réalisé le test de l'anticorps anti-HBe qui témoigne du contact certain avec le virus. Nous avons eu 19 cas de porteurs d´anticorps anti-HBc isolé la présence de cet anticorps qui ne procure aucune protection ou immunité contre le VHB pouvait s'interpréter de plusieurs manières: a) fenêtre biologique entre la disparition de l'AgHBs et l´apparition d´anti-HBs après une infection aiguë (personne infectée en voie de guérison); b) persistance de l´anticorps anti-HBc chez des personnes anciennement infectées; c) porteurs du VHB avec des niveaux d´AgHBs trop faibles pour être détectés par le test utilisé (personne infectée); d) enfin, un résultat biologique faussement positif (personne à risque). La présence de l'anticorps anti-HBc varie de 0,1% à 20% selon les populations [26,27]. Ainsi, la vaccination systématique pourrait être offerte à ces personnes et d'après McMahon *et al*., la majorité d'entre elles auront une réponse immunitaire primaire plutôt qu´une réponse de rappel au vaccin contre l'hépatite B [28]. Dans notre étude, nous avons alors retenu de vacciner tous les sujets ayant l'anticorps Anti-HBc isolé. Ce qui portait à 115 le nombre de participants à vacciner contre les 96 HSH en besoin réel de vaccination. Sur les 115 participants devant être vaccinés, 67 ont complété les trois doses de vaccin, 30 ont reçu deux doses et les 18 restants ont reçu une seule dose. Selon les résultats sur la recherche des anticorps, seulement 31/204 participants (15,2%), avaient uniquement l´anticorps anti-HBs sans l'anticorps anti-HBc qui témoigne d´un contact certain avec le virus. Ainsi, on peut estimer que ce sont seulement 15,2% de nos participants qui ont réellement été vaccinés; une couverture vaccinale relativement faible. De telle préoccupation a été rapportée dans plusieurs d'autres études nécessitant un plaidoyer auprès des autoritaires sanitaires nationales pour l´implantation d'un programme de dépistage systématique et de vaccination contre l´infection au VHB dans cette population supposée à haut risque [17,29-31].

Les facteurs significativement associés au portage de l´AgHBs et l´antécédent d´hépatite B étaient l´âge, les rapports sexuels sous l'effet de la drogue ou de l'alcool et le fait de vivre en couple avec le partenaire sexuel. Loukabou *et al*. en 2019 ont retrouvé l´âge au premier rapport sexuel, le non usage régulier du préservatif comme des facteurs associés à l´hépatite B à Brazzaville et à Pointe-Noire en république du Congo [21]. L´association avec l´âge dans notre étude pouvait s´expliquer par la distribution de l'infection selon l'âge où la prévalence était significativement plus élevée chez les plus âgés. Elle est semblable à l´association avec l´âge au premier rapport sexuel anal tel le cas en République du Congo. Les partenaires sexuels vivant en couple n´utilisent pas généralement de préservatif, et l'irrégularité d´usage de préservatif comme facteur associé retrouvé à Brazzaville et à Pointe-Noire est similaire à cette association avec la vie en couple dans notre étude. Les rapports sexuels sous l´effet de drogue ou d´alcool constituent un facteur généralement associé à la transmission des infections sexuellement transmissibles. Ce comportement est une composante des comportements à risques diversifiés des infections sexuellement transmissibles comme c'était le cas chez les hommes conducteurs de taxi-moto à Parakou en 2022, la seconde grande ville du Bénin après Cotonou, la capitale économique [32]. Il est à remarquer que la prévalence de l´hépatite C était très faible. Cette faible prévalence pourrait s'expliquer par la moindre prise de risque de transmission parentérale tels l'usage de drogue par voie intraveineuse, le sniff ou la consommation de crack qui sont majoritairement les voies de transmission de l´hépatite C [9].

Notre étude présente certaines limites. Nous n´avons pas fait la charge virage chez les sujets positifs au AgHBs et chez ceux uniquement positifs à l´anticorps anti-HBc. Ce qui nous a limité dans les interprétations de l´évolution de l'infection. Nous n'avons pas pu collecter de données pour mieux documenter l'hépatite C, car il s'agissait d´utilisation des données secondaires issues de l´étude de démonstration de la prophylaxie préexposition à laquelle l´infection au VHC était un critère de non-inclusion. Ainsi, les données étaient manquantes pour l'identification des facteurs associés à l´hépatite C. Nous n´avons identifié les facteurs associés que pour l´infection au virus de l´hépatite B. Nos prévalences estimées étaient en fait celles au sein de HSH séronégatifs pour le VIH qui pourraient sous-estimer les vraies prévalences paramétriques populationnelles des hépatites B et C de cette cible des HSH. L´échantillonnage était aléatoire à deux degrés, et le deuxième degré était par le parcours aléatoire. La cible étant inégalement répartie dans les quartiers de localisations reconnues seulement par les pairs-éducateurs dits animateurs-experts, il était possible d´avoir un biais d´itinéraire de convenance initié par ces pairs. Toutefois, même si un tel biais devrait survenir, il n'aurait pas été de nature à engendrer un biais de sélection différentiel pouvant invalider les facteurs associés. Les collectes de données ont été numériques et les agents collecteurs bien formés, on ne devrait pas craindre un biais d´information différentiel. Comme dans la plupart des études, la confondance résiduelle est possible. Cependant, ne s'agissant pas d´un modèle explicatif, de tels facteurs de confusion n´auraient pas fait craindre de problème de validité de mesure d'association.

## Conclusion

Le VHB est fréquent dans la population des HSH à Cotonou, particulièrement chez les HSH plus âgés qui sont plus susceptibles d´avoir commencé leur vie sexuelle avant le début des interventions spécifiques aux HSH au Bénin (2008). La prévalence du VHC était faible, probablement en raison de l'absence de consommation de drogues injectables et du fait que tous les participants étaient séronégatifs. Le dépistage et la vaccination contre le VHB devraient être systématiquement offerts aux HSH.

### Etat des connaissances sur le sujet

- *Les hépatites B et C sont considérées par l´OMS comme la quatrième priorité de santé publique à l´échelle mondiale;*
- *Les HSH courent un risque accru d´hépatites B et C s´ils adoptent des comportements favorisant la transmission parentérale;*
- *En raison des facteurs de risque partagés pour l´acquisition de virus, les HSH séropositifs pour le VIH courent un risque accru de contracter les virus de l´hépatite B et C*.

### Contribution de notre étude à la connaissance

- *À Cotonou, les HSH non infectés par le VIH ont une prévalence des hépatites B et C semblables à celles de la population générale du Bénin;*
- *La prévalence du portage d´AgHBs et celle de l´antécédent de l´hépatite B étaient plus élevée au sein des HSH âgés de 30 ans et plus comparativement aux plus jeunes;*
- *L´âge ≥30 ans, les rapports sexuels sous l´effet de la drogue ou de l´alcool et le fait de vivre en couple étaient les facteurs associés à l´hépatite B chez les HSH non infectés par le VIH*.
